# The Association of Mean Plasma Glucose and In hospital Death Proportion: A Retrospective, Cohort Study of 162,169 In-Patient Data

**DOI:** 10.1155/2021/1513683

**Published:** 2021-01-12

**Authors:** Peili Chen, Lili Chen, Xiaolong Zhao, Quanya Sun

**Affiliations:** Department of Endocrinology, Huashan Hospital Fudan University, Shanghai, China

## Abstract

**Aims:**

To investigate the association between mean plasma glucose and inhospital death proportion.

**Methods:**

We retrospectively collected 162,169 inpatient data in Huashan Hospital from January 2012 to December 2015. Mean plasma glucose was calculated and considered as the average glycemia control during hospitalization. Patients were stratified into six groups according to mean plasma glucose. Nonlinear regression was performed to determine the associations between mean plasma glucose and inhospital death proportion, medical cost, and length of stay. Multivariate logistic regressions were performed to evaluate the relationship of mean plasma glucose and outcomes controlling for confounders including age, gender, and others. Subgroup analyses were performed on basis of whether they were surgical patients, ICU patients, patients with diabetes, or others.

**Results:**

Of the 162,169 hospitalized participants, 53.32% were male and 989 died during hospitalization. Nonlinear regression showed there were positive and significant associations between mean plasma glucose and death proportion, medical cost, and length of stay (*P* < 0.001 for all). Multivariate logistic regressions showed that, compared with group B, a statistically significant association between mean plasma glucose and predicted outcome was apparent, with the odds ratios (95% confidence interval) of 5.79 (3.51–9.55), 2.85 (2.40–3.38), 6.29 (5.24–7.54), 9.34 (7.51–11.62), and 23.52 (16.64–33.26), for group A, group C, group D, group E, and group F, respectively. There was a U-shaped association between mean plasma glucose and death proportion. Subgroup analyses showed similar associations between mean plasma glucose and death proportion, medical cost, and length of stay as in the whole sample.

**Conclusions:**

There was a U-curve association between mean plasma glucose with inhospital death proportion. Mean plasma glucose was associated positively with medical cost and length of stay.

## 1. Introduction

There is growing awareness that diabetes has become an epidemic disease and glucose control in hospital is important for the clinical outcomes, especially among the individuals with diabetes. In China, the recent national survey in 2013 reported that the prevalence of diabetes and prediabetes was 11.6% and 50.1%, respectively, accounting for 113.9 million adults with diabetes and 493.4 million adults with prediabetes [1]. Hence, the proportion of dysglycemia in hospital is inevitably increasing. Up to 25–30% of the patients in adult wards and critical care units in American hospitals had diabetes [2]. Similar Chinese studies have shown that the prevalence of diabetes in hospitalized patients was 15.7%, with a prevalence of prediabetes of 21.9% [3]. Many studies demonstrated that hyperglycemia was associated with mortality and morbidity in hospitalized patients [4–8]. One retrospective observational study showed that adverse postoperative events increasing by 30% were associated with intraoperative glucose level increasing by every 20 mg/dL (1.1 mmol/L) [9]. A prospective, randomized, controlled study in ICU patients found that if blood glucose was maintained between 4.4 and 6.1 mmol/L, the inhospital mortality would be reduced by 34%, the bloodstream infections would be reduced by 46%, and acute renal failure requiring dialysis or hemofiltration would be reduced by 41% [10, 11]. Another research suggested that if the mean glucose value was lowered from 8.5 mmol/L (152.3 mg/dL) to 7.3 mmol/L (130.7 mg/dL), the inhospital mortality would decrease by 29.3%, and length of stay in ICU would decrease by 10.8% [12–14]. Therefore, more and more attention has been paid to the management of blood glucose with the collection of clinical evidence of bettering inpatient glucose management, particularly in the western world. However, the practice of inpatient glucose management in China just emerged and was questioned recently, due to the different models of the traditional Chinese and Western inhospital blood glucose management. Most of the patients with diabetes in the western countries are followed up in the outpatient clinic, while these in China are used to hospitalization. Insulin regimen of the inpatients in China is mainly premixed insulin twice a day, while in western countries, it is mainly basal and bolus insulin program [15]. The lack of information technology integrated hospital glucose management is also the reason for hindering its development. The core problem is the lack of Chinese evidence to elucidate the association between inhospital blood glucose levels and mortality. So, we examined the relationship between mean plasma glucose and inhospital death proportion, medical cost, and length of stay.

## 2. Methods

We retrospectively collected the information of adult patients admitted to Huashan Hospital Fudan University from January 1, 2012, to December 31, 2015. Huashan Hospital is a large teaching hospital of approximately 1200 beds with a full range of adult tertiary care services. When admitting hospital, all patients signed an informed consent form that their clinical data may be used in clinical research. We extracted the clinical data through hospital coding and e-discharge summary systems, including department category, gender, age, admission date, date of discharge, hospital admission number, medical cost, inhospital death, the way of discharge, and all plasma glucose records during hospitalization from the information center. The personal privacy information including the patient's name and identity number was not included and technically protected according to the patient protection rules on electronic record information set by the Huashan Hospital ethics committee and department of information. The arterial blood glucose measures and capillary blood glucose measures were not included. Patients who were lacking electronic records of clinical data and did not have at least one plasma glucose measure during hospital were excluded.

We defined diabetes as the presence of International Classification of Diseases, Ninth Revision, Clinical Modification (ICD-9-CM) codes 250.xx in the index or prior admission discharge abstract [16]. All plasma glucose values were included, including admission glucose values and fasting and nonfasting glucose values. Mean plasma glucose for each patient was calculated from the average of all plasma glucose values measured during the hospital [17]. The plasma glucose measurements used in the analysis were measured using the glucose oxidase method in the Johnson VITROS 5.1FS biochemistry analyzer. The length of stay was calculated through the day of admission and discharge.

Patients were stratified into six categorical groups of mean glucose based on the distribution of data and findings from a prior study [13]: group A: ≤3.9 mmol/L, group B: 3.9–6.1 mmol/L, group C: 6.1–8.0 mmol/L, group D: 8.0–11.1 mmol/L, group E: 11.1–16.7 mmol/L, and group F: >16.7 mmol/L. Subgroup analyses were performed on the basis of whether or not they were surgical patients, patients in ICU, and patients with diabetes. Our study was conducted in accordance with the ethical principles of the Declaration of Helsinki and with approval from the Ethics Committee of Huashan Hospital.

### 2.1. Statistical Analysis

Normally distributed data were expressed as means ± SD, whereas variables with a skewed distribution were reported as median (interquartile range) and log-transformed to approximate normality before analysis. Categorical variables were represented by frequency and percentage. The mean plasma glucose was categorized into six groups and group B was set as the reference. The death proportion was calculated with the number of dead patients divided by the total number of patients in a certain group. Then, we used a quadratic regression equation to fit the changing trend of death proportion. Nonlinear regression was performed to determine the associations between mean plasma glucose and inhospital death proportion, medical cost, and length of stay. Multivariate logistic regressions have been employed to evaluate the relationship of mean plasma glucose and predicted outcome (death proportion) controlling for confounder factors such as age gender. Further subgroup analyses were examined using the nonparametric Spearman test. *P* value <0.05 was considered statistically significant. All statistical analyses were conducted using the SPSS 23.0 system.

## 3. Results

### 3.1. Study Participants

The general characteristics of the study participants are presented in Table 1. The enrolled 162,169 subjects in our study were selected from a total of 250,000 consecutive admissions to Huashan Hospital during a 3-year period from January 2012 to December 2015. In total, 14,188 (8.75%) participants had a history of diabetes. There were 53.32% male participants and the median age was 54 (41–56) years. The median medical cost was 1917.3 (970.4–4969.3) US dollar. The median length of stay was 10.2 (4.5–13.5) days. 28.21% of the cohort was admitted to the surgical ward and 3.37% were in the ICU. There were statistical differences in death proportion if grouping according to gender, age, mean glucose, ICU/non-ICU, surgical/nonsurgical, and diabetes/nondiabetes. Inhospital death proportion for each group (Table 2) was 1.06% (group A), 0.23% (group B), 1.02% (group C), 2.62% (group D), 4.53% (group E), and 8.01% (group F). The inhospital death proportion was 1.70% in ICU patients, 1.94% in surgical patients, and 1.41% in patients with diabetes. The death proportion during admission was 0.6%. The majority of the mean hospitalization glucose level was in 5-6 mmol/L range (Supplementary Figure 1).

### 3.2. Association between Mean Plasma Glucose Levels and Inhospital Death Proportion

We investigated the association between mean plasma glucose and the death proportion, medical cost, and length of stay through the nonlinear regression (*P* < 0.001 for all). After calculating the inhospital death proportion, we used the quadratic regression equation to fit the changing trend of death proportion. We found that there was a U-curve association between mean plasma glucose and inhospital death proportion (Table 2). The death proportion was the lowest when the mean glucose was in group B (3.9–6.1 mmol/L).

Then, we assessed the association of mean plasma glucose levels stratified in six groups on length of stay and medical cost. Compared with group B, a statistically significant association between mean plasma glucose and medical cost was apparent (*P* < 0.001). When comparing with group B, there was a positive association between mean plasma glucose and the length of stay (*P* < 0.001) in all groups, besides group A. With the mean plasma glucose increasing, the length of stay was longer and the medical cost was more, especially in group E (Table 2).

Multivariate logistic regressions have been employed to evaluate the relationship of the mean plasma blood glucose status and predicted outcome (death proportion) controlling for confounder factors such as age and gender (Table 3). We found that compared with group B (mean plasma glucose, 3.9–6.1 mmol/L), a statistically significant association between mean plasma glucose and predicted outcome was apparent, with the odds ratios (95% confidence interval) of 5.79 (3.51–9.55), 2.85 (2.40–3.38), 6.29 (5.24–7.54), 9.34 (7.51–11.62), and 23.52 (16.64–33.26), as for groups with mean plasma glucose ≤3.9 mmol/L, 6.1–8.0 mmol/L, 8.0–11.1 mmol/L, 11.1–16.7 mmol/L, and >16.7 mmol/L, respectively.

### 3.3. Subgroup Analyses: Association between Mean Plasma Glucose and Clinical Outcomes

**Figure 1:**
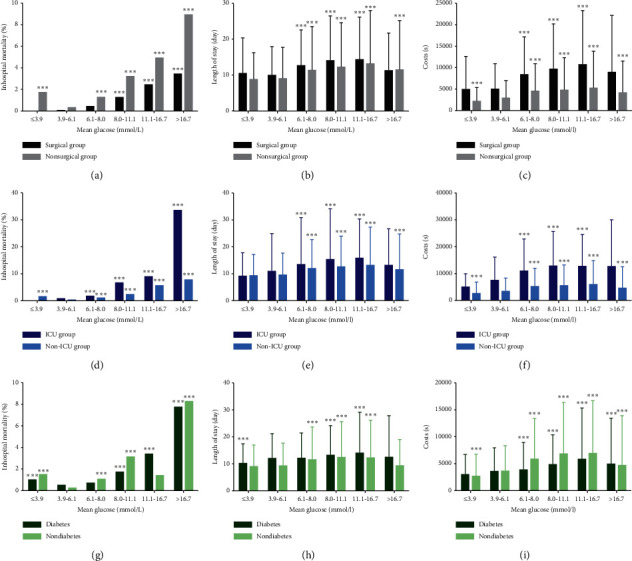
Subgroup analyses of the associations between mean plasma glucose and clinical outcome. (a–c) Correlation of inhospital death proportion (a), hospitalization days (b), and medical costs (c) with mean plasma glucose in further surgical/nonsurgical subgroup analyses. (d–f) Correlation of inhospital death proportion (d), hospitalization days (e), and medical costs (f) with mean plasma glucose in further ICU/non-ICU subgroup analyses. (g–i) Correlation of inhospital death proportion (g), hospitalization days (h), and medical costs (i) with mean plasma glucose in further diabetes/nondiabetes subgroup analyses. ^*∗∗∗*^: *P* < 0.001, compared with normoglycemia (3.9–6.1 mmol/L) group. ICU, intensive care unit.

Further subgroup analyses were performed on the basis of whether they were surgical, ICU, or patients with diabetes and group B was set as reference (Figure 1). In both surgical and nonsurgical patients, the death proportion increased as the mean plasma glucose rose, except for group A (Figure 1(a)). In nonsurgical patients, there was a positive association between mean plasma glucose and the length of stay (Figure 1(b)) in all groups excluding group A. In surgical patients, the positive association between mean plasma glucose and length of stay was found in all groups excluding groups A and F (Figure 1(b)). The association between mean plasma glucose and medical cost was found in all groups for nonsurgical patients. The similar association between mean plasma glucose and medical cost was found in all groups excluding groups A and F for surgical patients (Figure 1(c)). For ICU patients, the length of stay (Figure 1(e)) and medical cost (Figure 1(f)) were associated with the mean plasma in all groups except for groups A and F. For non-ICU patients, the length of stay was associated with mean plasma in all groups except for group A (Figure 1(e)), while the medical cost was associated with mean plasma in all groups (Figure 1(f)). For patients with diabetes, their inhospital death proportion increased as the mean plasma glucose rose, except for group A (Figure 1(g)). The death proportion of patients without diabetes showed a similar trend. In patients with diabetes, there was a positive association between mean plasma glucose and the length of stay (Figure 1(h)) in all groups excluding group F. Similarly, in patients with diabetes, mean plasma glucose was associated with medical cost in all groups, besides group A (Figure 1(i)). In patients without diabetes, mean plasma glucose was positively associated with medical cost in all groups (Figure 1(i)) and was positively associated with length of stay in all groups excluding groups A and F (Figure 1(h)).

### 3.4. Comparison of Clinical Outcomes between Different Subgroups

Surgical patients have significantly higher medical costs (Figure 1(c)) and a lower death proportion (Figure 1(a)) than nonsurgical patients in groups A, B, C, D, E, and F (all *P* < 0.05). Surgical patients have a significantly longer length of stay (Figure 1(b)) than nonsurgical patients in groups A and D (both *P* < 0.05).

Patients in ICU have a significantly higher death proportion (Figure 1(d)) and longer length of stay (Figure 1(e)) than those in general wards in groups B, C, D, E, and F (all *P* < 0.05). Patients in ICU have significantly more medical costs (Figure 1(f)) than those in general wards in groups A, B, C, D, E, and F (all *P* < 0.05).

Patients with diabetes have a significantly higher death proportion (Figure 1(g)) than those without diabetes in groups B and E (both *P* < 0.05). Patients with diabetes have a significantly longer length of stay (Figure 1(h)) than those without diabetes in groups B, E, and F (all *P* < 0.05). The differences in the medical cost between patients with and without diabetes (Figure 1(i)) do not reach statistical significance in groups A, B, C, D, E, and F (all *P* > 0.05).

## 4. Discussion

### 4.1. Statement of Principal Findings

In this retrospective study of 162,169 medical patients, we have shown that there was a U-shaped association between mean glucose with inhospital death proportion. In addition, mean plasma glucose was positively associated with medical costs and length of stay. In subgroup analyses, we found that these associations were still significant, regardless of the severity of illness, surgical intervention, or concomitance with diabetes. These findings support the results of previous studies from other settings and different patient populations focusing on glycemic condition and mortality [18, 19]. According to subgroup analyses, we also confirmed that patients with diabetes have a longer length of stay than patients without diabetes; patients in ICU also have a longer length of stay and more medical costs than those in general wards; surgical patients have a longer length of stay and more medical costs than those without surgery. The findings of subgroup analyses are also consistent with previous studies [5, 13, 20, 21]. Several previous research paid attention to admission glucose [5, 11, 14, 22]. Our study has focused on mean plasma glucose. A previous study compared the predictive value of 3 different glucose measurements (mean glucose, time-averaged glucose, and hyperglycemic index) over 3 time windows (first 24 hours, 48 hours, entire hospitalization) for inhospital mortality with that of admission glucose. It concluded that mean glucose during the entire hospitalization appeared to be the most practical metric for hyperglycemia-associated risk [17]. Hence, in our study, mean hospitalization glucose was calculated to conduct research and analysis. And we also found on the U-shaped curve that the lowest death proportion was for mean glucose between 3.9 and 6.1 mmol/L. It may be because most plasma glucose data are fasting plasma glucose, accounting for 98.5%. Therefore, the mean plasma glucose could be considered as a good indicator of the average glycemia control during hospitalization.

We have shown that there was a U-shaped association between mean glucose with inhospital death proportion. Our findings support the results of previous studies focusing on inhospital glucose and mortality [23, 24]. Falciglia et al. [13] showed that hyperglycemia was a potentially harmful and correctable abnormality in critically ill patients and was associated with increased mortality independent of intensive care unit type, length of stay, and diabetes. Another study showed that the lowest hospital mortality, 9.6%, occurred among patients with mean glucose values between 80 and 99  mg/dL ((4.4–5.5) mmol/L), and as glucose values increased, hospital mortality increased progressively. When mean glucose values exceeded 300 mg/dL (16.6 mmol/L), inhospital mortality reached 42.5% [24]. Most researchers focused on patients with hyperglycemia or acutely and critically ill patients. We focused on patients with normal glucose or abnormal glucose and also performed subgroup analyses to verify the results.

Why hypoglycemia and hyperglycemia are associated with a higher inhospital death proportion? Hypoglycemia leads to insufficient organ energy metabolism and organ dysfunction. Hypoglycemia increases the risk of thrombosis and inflammation and may induce metabolic encephalopathy and arrhythmia [25–27]. Hyperglycemia inhibits the phagocytosis of inflammatory cells and impairs the immune response, which would induce or aggravate nosocomial infection [28]. Hyperglycemia may also delay wound healing, impair cardiac metabolism, decrease glomerular filtration rate, and impair endothelial function [29, 30]. All of these factors contribute to the increased death proportion.

### 4.2. Weaknesses and Future Research

In the current study, we used the clinical data center to collect medical records and match with clinical laboratory data. Hence, it may reduce human error in data collection, avoid cumbersome data entry, and save time. Our study conclusion was similar to previous studies. However, due to the data records being incomplete, it was impossible to use other laboratory indicators, severity of illness, treatment of diabetes, infection, and the follow-up of patients to add more measurements in multivariate logistic regressions. And due to laking the date about the frequency of venous blood glucose measurement, it is impossible for us to explore the relationships between the MAGE, FPG-CV, and MODD with outcomes. In the future, we may conduct a multicenter prospective controlled research and expand the sample size.

## 5. Conclusions

In this retrospective cohort study, we evaluated the association between mean glucose and death proportion as well as the length of stay and medical cost. Our findings suggested a U-curve association between mean plasma glucose and inhospital death proportion. It may imply that all patients admitted to the hospital should have their blood glucose level checked and monitored during their stay to control in a proper range, avoiding poor outcomes from hypoglycemia and hyperglycemia.

## Figures and Tables

**Table 1:** Characteristics of the study population.

	*n* (%)	Inhospital death, *n* (%)	*P*
*Gender* ^*∗*^			<0.001
Male	86475 (53.32)	672 (0.78)	
Female	75694 (46.68)	317 (0.42)	

*Age* ^*∗*^ *(y)*			<0.001
≤17	3153 (1.94)	4 (0.12)	
18–40	37603 (23.19)	57 (0.15)	
41–65	84015 (51.80)	291 (0.35)	
66–85	32103 (19.80)	420 (1.30)	
≥86	5295 (3.27)	217 (4.10)	

*Mean glucose* ^*∗*^ *(mmol/L)*			<0.001
≤3.9	1794 (1.11)	19 (1.06)	
3.9–6.1	121285 (74.79)	280 (0.23)	
6.1–8.0	26521 (16.35)	271 (1.02)	
8.0–11.1	8860 (5.46)	232 (2.62)	
11.1–16.7	3160 (1.95)	143 (4.53)	
>16.7	549 (0.34)	44 (8.01)	

Non-ICU^*∗*^	156703 (96.63)	896 (0.57)	<0.001
ICU^*∗*^	5467 (3.37)	93 (1.70)	

Nonsurgical^*∗*^	116429 (71.79)	100 (0.09)	<0.001
Surgical^*∗*^	45740 (28.21)	889 (1.94)	

Nondiabetes^*∗*^	147981 (91.25)	789 (0.53)	<0.001
Diabetes^*∗*^	14188 (8.75)	200 (1.41)	

In total	162 169	989 (0.60)	

^*∗*^ *P* < 0.001. ICU, intensive care unit.

**Table 2:** Association of mean glucose with in hospital death proportion, medical cost, and length of stay.

	Mean glucose (mmol/L)							
	≤3.9	3.9–6.1	6.1–8.0	8.0–11.1	11.1–16.7	>16.7	Total	*P*
Age (y)^*∗*^	1794	121285	26521	8860	3160	549	162169	
	50.50 (34, 64)	52 (38, 63)	59 (49, 68)	61 (52, 70)	61 (53, 70)	61 (52, 72)	54 (40, 65)	<0.001
Male gender (%)	47.80	52.50	55.60	56.40	58.30	59.00	53.30	0.02
Length of stay ^*∗*^ (d)	7 (4.50, 11.50)	7.50 (4.50, 12)	9 (4.50, 15.50)	10 (5, 17)	10 (5.50, 17)	8.5 (4.50, 14.50)	10.20 (4.50–13.50)	<0.001
Medical cost ^*∗*^ (hundred USD)	14.50 (7.00, 27.40)	17.20 (9.20, 41.30)	28.20 (11.90, 77.40)	32.10 (13.40, 82.40)	30.70 (13.60, 78.10)	22.80 (10.50, 52.60)	1917.30 (970.40–4969.30)	<0.001
Inhospital death ^*∗*^ (*n*, %)	19 (1.06)	280 (0.23)	271 (1.02)	232 (2.62)	143 (4.53)	44 (8.01)	989 (0.60)	<0.001

^*∗*^ *P* < 0.01. *P* values were determined using Student's *t* test, the nonparametric Spearman test, or the chi-square test, if appropriate.

**Table 3:** Multivariate logistic regression analysis to include mean plasma glucose for prediction of death proportion.

Group^*∗*^	Beta	SE	*P* value	OR	95% CI for OR
Group A	1.76	0.25	<0.001	5.79	3.51–9.55
Group C	1.05	0.08	<0.001	2.85	2.40–3.38
Group D	1.84	0.09	<0.001	6.29	5.24–7.54
Group E	2.23	0.11	<0.001	9.34	7.51–11.62
Group F	3.16	0.18	<0.001	23.52	16.64–33.26

^*∗*^N*ote*. Mean PBG was categorized into six groups: group A: ≤3.9 mmol/L, group B: 3.9–6.1 mmol/L, group C: 6.1–8.0 mmol/L, group D: 8.0–11.1 mmol/L, group E: 11.1–16.7 mmol/L, and group F: >16.7 mmol/L. Group B was set as the reference. The multivariate logistic regression model adjusted for age, gender, length of days, ICU, diabetes/nondiabetes, and surgery/nonsurgery. ICU, intensive care unit.

## Data Availability

Due to ethical concerns and because the original data involves the privacy of the patients, it is not suitable for publication. The original data can be obtained from the corresponding author upon reqeust.

## Additional Points

- The concept of inhospital blood glucose management has not been popularized in China, due to a lack of Chinese evidence.
- We collected the clinical data including mean glucose levels in a medical cohort of 162,169 inpatients to determine the association of mean plasma glucose and inhospital death proportion.
- In our study, we used mean plasma glucose as the average glycemia control measurement during hospitalization and proved that mean plasma glucose and inhospital death proportion were U-shape associated in China. Moreover, mean plasma glucose was correlated positively with medical cost and length of stay. We further made subgroup analyses according to whether or not they were surgical patients, patients in the intensive care unit (ICU), and patients with diabetes. On subgroup analyses, we found similar associations.

## Supplementary Materials

Supplementary Materials Supplementary Figure 1: cumulative frequency curve of inhospital mean glucose.
