# Supplementary figures and images for: The Association of Mean Plasma Glucose and In hospital Death Proportion: A Retrospective, Cohort Study of 162,169 In-Patient Data
Source: Int J Endocrinol. 2021 Jan 12;2021:1513683. doi: 10.1155/2021/1513683 (PMC7834774; doi:10.1155/2021/1513683)

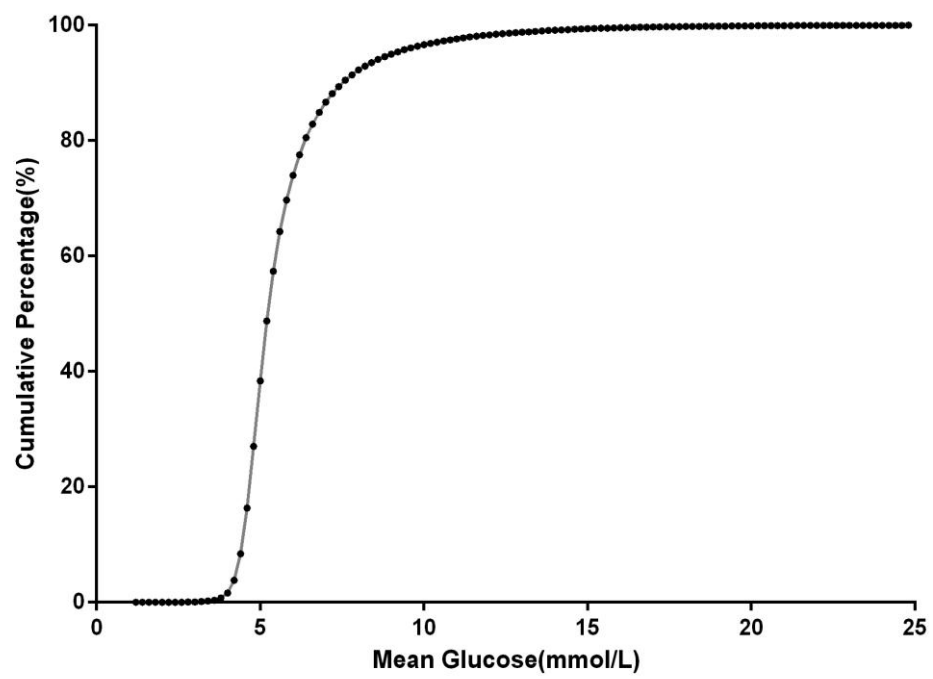

Supplementary Figure 1 Cumulative frequency curve of in-hospital mean glucose

Supplement: Supplementary Materials — Supplementary Figure 1: cumulative frequency curve of inhospital mean glucose. [file 1513683.f1.pdf]
